# From strain to strength through humor: resilience in professional football players

**DOI:** 10.3389/fpsyg.2025.1699013

**Published:** 2026-01-05

**Authors:** Aydın Pekel, Mehmet Behzat Turan, Vesile Şahiner Güler, Mujahid Iqbal, Osman Pepe, Abdullah Arısoy, İbrahim Dalbudak

**Affiliations:** 1 Department of Sports Management, Faculty of Sports Sciences, Marmara University, İstanbul, Türkiye; 2 Department of Recreation, Faculty of Sports Sciences, Erciyes University, Kayseri, Türkiye; 3 Department of Physical Education and Sports, Institute of Health Sciences, Erciyes University, Kayseri, Türkiye; 4 Beijing Key Laboratory of Applied Experimental Psychology, Faculty of Psychology, National Demonstration Center for Experi-10 Mental Psychology Education (Beijing Normal University), Beijing, China; 5 Department of Sports Management, Faculty of Sports Sciences, Süleyman Demirel University, Isparta, Türkiye; 6 Süleyman Demirel University, Faculty of Sports Sciences, Uşak University, Isparta, Türkiye; 7 Department of Sports Management, Faculty of Sports Sciences, Uşak, Türkiye

**Keywords:** psychological strain, coping humor, mental well-being, football players, sport psychology

## Abstract

**Background:**

Athletes often face competitive stress, with resilience and coping humor as key resources to mitigate its negative impact on mental well-being and performance.

**Objective:**

This study aims to examine the relationships between psychological strain, resilience, and coping humor among professional Turkish football players, and to investigate the moderating role of humor-based coping in the link between strain and resilience.

**Method:**

This cross-sectional study involved 466 professional Turkish football players (2024–2025 season). Data were collected via the APSQ, BRS, and CHS, and analyzed using SPSS v22, which included correlations, regressions, and moderation testing with the PROCESS Macro Model 1 (5,000 bootstraps). Additionally, the Jamovi 2.6.2.0 Package program was used.

**Results:**

Psychological strain significantly predicted resilience, and coping humor showed a direct positive effect on resilience. Significantly, coping humor moderated the relationship between strain and resilience, such that the positive association was strongest at high levels of coping humor. Athletes with higher humor-based coping skills were likelier to transform psychological strain into resilience, while the effect was nonsignificant at low humor levels.

**Conclusion:**

The findings demonstrate that coping with humor strengthens athletes’ resilience and amplifies the beneficial effects of psychological strain when interpreted as a challenge. Integrating humor-based interventions into psychological training programs may enhance football players’ adaptive coping skills, resilience, and overall mental well-being.

## 1 Introduction

Individuals often encounter both positive and negative experiences in their daily lives. The capacity to cope with adversity and the development of effective coping strategies are related to an individual’s general well-being. Individuals employ various coping strategies to manage the challenges they encounter in their lives. These strategies may vary depending on the social and physical environment in which the individual lives, especially in sports environments where intense competition is prevalent; these coping strategies may be more pronounced in such settings. Depending on an individual’s appraisal and coping approach, stress is a primary factor leading to declines in athletic performance (McGreary et al., 2020).

Considering the competitive environment in sports, it is essential to remember that this environment can have both positive and negative impacts on athletes as they strive for excellence. Jones et al. (2009) developed and later revised the theory of challenge and threat situations in athletes to explain how athletes anticipate and prepare for motivated performance situations (e.g., sporting competitions). This theory focuses on the physiological and emotional responses to stress and their impact on sports performance. It was stated that athletes respond to competition in the following ways: challenge and threat. In this theory, when athletes respond positively to stress in competitive environments, it creates a feeling of “challenge.” When they respond negatively, it creates a feeling of “threat.” Challenge and threat are two different psychophysiological responses to stressors (Jones et al., 2009; Meijen et al., 2020). Therefore, how the athlete perceives and reacts to stress can directly affect psychological and physical performance. The mental and physical reactions during competition should be evaluated individually, not independently of each other. It is thought that this magnitude affects the mental well-being of athletes.

The World Health Organization (2004) defined mental well-being as “being aware of one’s abilities, overcoming the stress in one’s life, being productive and useful in business life, and contributing to society in line with one’s abilities” (World Health Organization, 2004). It has been stated that stress, which is expressed as one of the determinants of success in sports, plays an effective role in athletes’ personal psychological resilience and coping strategies, management, reduction, recovery, and ability to quickly return to their everyday lives (Nicholls et al., 2009; Codonhato et al., 2018a; Cappella and Rhona, 2001). At this point, it is believed that the feelings of challenge and threat exhibited by athletes in response to the psychological and physiological pressures they face are related to their mental well-being.

In recent years, studies in sport psychology have increasingly emphasized the role of athletes in regulating stress and developing adaptive coping mechanisms under pressure (Nuetzel, 2023; Nogueira et al., 2025). Empirical findings suggest that humor and positive reframing are among the most effective psychological tools for managing performance-related stress and maintaining motivation (Dakwo et al., 2023). These findings underscore the importance of incorporating emotion regulation and humor-based coping into contemporary models of resilience in sport psychology.

Athletes’ family background and personal goals can influence how they perceive stress and choose humor as a coping mechanism, shaping both resilience and performance outcomes (Coulter et al., 2018; Tian et al., 2022).

During competitive events, athletes must exhibit a range of abilities to cope with psychological pressure and stress, as well as to maintain their strength, endurance, and ability to resist fatigue throughout the competition (Fauvel and Ducher, 2009; Christian et al., 2013; Sarkar and Fletcher, 2014). Adaptive humor styles have been shown to buffer anxiety and distress during performance, suggesting that cultural, environmental, and individual factors jointly influence how athletes cope with stress (Dakwo et al., 2023; Munir and Pandin, 2020).

However, despite the growing body of research on stress and resilience in sports, the role of humor as a moderating coping mechanism remains underexplored, particularly in competitive team contexts such as football. Recent systematic reviews indicate that few empirical studies have examined how humor facilitates psychological adaptation and team cohesion in elite sports (Simon et al., 2024; Nuetzel, 2023). This study aims to address this research gap by empirically testing the moderating role of coping humor in the relationship between psychological strain and resilience among football players.

This study conceptualizes psychological strain as the independent variable (IV) that negatively influences mental health outcomes. Psychological resilience, defined as the capacity to recover from adversity, is treated as the dependent variable (DV). Coping humor is a moderator, reflecting how individuals manage stress and maintain adaptive functioning. Environmental context and cultural factors, including team culture and societal norms, further shape coping strategies, reinforcing the conceptual link between psychological strain, resilience, and humor as a coping strategy (Nagy and Molnárné, 2022; Munir and Pandin, 2020).

### 1.1 Psychological strain

Stress, also known as psychological strain or tension, is a process that leads to psychological and biological emotional tension when a person cannot manage his/her current situation in the process of adapting to expectations as a result of interaction with the environment (Gibbons, 2012; Abdel Wahed et al., 2017). The concept of psychological strain generally reflects the instantaneous stress levels of individuals or the feelings and thoughts they experience in a specific period and refers to the stress levels experienced by individuals regarding the events they experience; the more negative the feelings and thoughts of individuals regarding these events, the higher their stress levels (Shaw et al., 2017; Asıcı and Uygur, 2017). Individuals may exhibit stress reactions when they perceive themselves as lacking sufficient coping resources to manage the events they experience; similarly, stress-related psychological strain symptoms can occur in athletes when the current demands exceed their coping capacity (Raedeke and Smith, 2004; King and Beehr, 2017). Strain theory explains three primary sources of psychological strain: failure to achieve goals of positive value, eliminating positive stimuli, and encountering negative stimuli. These situations can trigger negative emotions such as anger, frustration, and hopelessness that can only be managed through effective coping mechanisms (Agnew, 1992).

In the sports domain, contemporary evidence confirms that psychological strain is closely linked with burnout, decreased motivation, and impaired performance outcomes (Qi, 2025; Stoyanova et al., 2025). These findings highlight the importance of being able to reframe stressors and manage perceived strain in sustaining optimal performance under competitive pressure.

In today’s world, athletes’ physical skills and abilities alone are insufficient for sporting success. In addition to physical skills and abilities, athletes’ psychological skills and emotions are crucial for achieving sporting success and optimal performance (Rubaltelli et al., 2018). In this context, it is reported that stress, which is an important determinant of success in sport, has an impact on athletes’ personal psychological resilience and coping strategies, their capacity to manage, reduce, and improve stress and to quickly resume their everyday lives (Nicholls et al., 2009; Codonhato et al., 2018a; Cappella and Rhona, 2001).

### 1.2 Psychological resilience

Resilience has been defined as the ability of individuals to survive and thrive despite encountering adverse conditions (Hollister-Wagner et al., 2001). Psychological resilience can be defined as the ability to cope with stress or negative situations and emerge from them successfully. This dynamic process involves the interaction of current life conditions and past life experiences, rather than innate personality traits (Meredith et al., 2011). Tugade et al. (2004) defined psychological resilience as the ability to respond to changing conditions and recover from negative emotional experiences.

To cope with stress, athletes need to enhance their psychological resilience, which encompasses concepts such as crisis management and overcoming challenges, by refining their mental skills (Galli and Gonzalez, 2015). Masten and Coatsworth (1998) reported that an individual’s ability to perceive various factors, such as high self-esteem, mental competence, optimism, agreeableness, humor, and experience, is related to an individual’s psychological resilience.

Recent empirical evidence further underscores that resilience in athletes supports emotion regulation, reduces burnout, and enhances long-term motivation (Stoyanova et al., 2025; Qi, 2025). It enables athletes to maintain confidence under pressure, recover from setbacks, and sustain high performance in demanding conditions. Thus, resilience functions as both a personal strength and a protective psychological mechanism within the athletic context.

### 1.3 Coping humor

As previously mentioned, the notion that humor can serve a comparable role in moderating stress is widely regarded as self-evident. Many psychological theorists have identified humor as an adaptive strategy for coping (Martin and Lefcourt, 1983). Humor is one of the positive features of positive psychology that facilitates people to cope with and adapt to challenging situations (Martin et al., 2003). The concept of coping humor has been defined as the behaviors that individuals with a positive outlook on life and strong psychological resilience (Celso et al., 2003) use to react to stressful or distressing situations they encounter in their daily lives (Lefcourt, 2001; Martin and Ford, 2018). These behaviors include emphasizing the humorous aspects of the situation and using humor, such as jokes, to reduce the stress associated with the situation (Yerlikaya, 2009; Chauvet and Hofmeyer, 2007).

The literature review conducted by researchers has shown that using humor is an effective method for problem-solving and coping with stress (Çelebi et al., 2021; Brunault et al., 2016; Vela et al., 2013; Satıcı and Deniz, 2017). At the same time, humor is believed to enhance the quality of life for individuals due to its positive effects on both psychological and physical health. Humor supports resistance and coping power, improves health and well-being, strengthens social relationships, and facilitates communication (Chauvet and Hofmeyer, 2007). Accordingly, humor can be seen not only as a way of relaxation in coping with stressful situations, but also as an effective coping strategy that contributes to the development of more flexible and adaptive responses to difficulties by increasing the psychological resilience of the individual.

Within sports, coping humor is increasingly recognized as a mechanism that enhances team morale, social cohesion, and adaptive functioning under pressure (Simon et al., 2024; Nogueira et al., 2025). Athletes who employ humor are better able to regulate emotions, maintain optimism, and foster supportive team dynamics, which ultimately contribute to collective resilience.

### 1.4 Present study

Despite the expectation that athletes will perform at the highest level in their chosen sport, several potential issues that may arise in the competitive environment may prevent them from doing so (Flett and Hewitt, 2005; Mellalieu et al., 2009). To date, various psychosocial factors, including mental health, hopelessness, and depression, self-image (e.g., Rice et al., 2020; Sun et al., 2020), have been linked to psychological strain in different athlete groups. Additionally, various psychosocial factors, including emotion, psychological well-being, stress, and recovery, as well as stress-coping strategies (e.g., Trigueros et al., 2019; Codonhato et al., 2018b; Küçük Kılıç, 2020), have been linked to psychological resilience in different athlete groups.

The reason for choosing football in this study is that football has evolved from being just a game into a multidimensional phenomenon that has created its industry and values. As a significant industry, the pursuit of victory both materially and spiritually is central to football. This focus can impose various physical and psychological pressures on football players. While previous studies on football players indicate that psychological factors influence performance during competition, the extent to which psychological factors can enhance the performance of football players is a topic of increasing interest in sports psychology (Morris, 2000; Trail et al., 2005; Abdullah et al., 2016; Olmedilla et al., 2019; Arısoy and Pepe, 2021; Kalinowski et al., 2020).

When the literature is examined in the context of challenge and threat theory, it is seen that there are limited studies related to the psychological performance of athletes. Williams and Cumming (2012) reported that athletes’ imagery is positively related to the perception of challenge and negatively related to the perception of threat. Cankurtaran (2021) reported that threat perceptions increase as athletes’ fear of performance failure increases. Moore et al. (2013) applied challenge manipulations to one and threat manipulations to the other during the golf swings of two groups of experienced golfers. They reported that the challenge group performed better than the threat group and that it is important to consider the effect of competitive pressure on motor performance. A review of research focusing on football players indicates that Türkyılmaz (2019) identified a statistically significant association whereby higher levels of mental toughness are linked to an increased perception of challenge and a decreased perception of threat. Pepe et al. (2025) reported that the theory of challenge and threat plays a mediating role between positive thinking skills and excellent performance perceptions among football players.

The selection of regional professional league footballers is important because this league is recognized as a professional league in Türkiye and is considered the final stage of transitioning to professional football. Therefore, it is believed that footballers’ psychological and physiological responses to the pressures they face in this league will impact their mental well-being and performance on the field. At this point, depending on the challenge and threat theory, humor is thought to play a significant role in helping footballers cope with the pressures that may occur both on and off the field.

In our literature review, no study examined the relationship between psychological strain, resilience, or coping humor in any athlete group. It is anticipated that the results obtained from this study will significantly contribute to the existing body of knowledge. As a preliminary step toward addressing these knowledge gaps, the objectives of this study were to (a) examine the direct effect of psychological strain on psychological resilience in football players and (b) explore the moderator effect of coping humor on the relationship between psychological strain and resilience in football players.

### 1.5 Theoretical framework and rationale for moderation

Moderation analysis is a statistical approach used to test whether the strength or direction of the relationship between an independent variable and a dependent variable depends on the level of a third variable (Hayes et al., 2017). In the present study, psychological strain was considered the independent variable (X), psychological resilience the dependent variable (Y), and coping humor the moderator (W). This framework is beneficial in psychological and sports sciences, where stress-outcome relationships are often contingent upon personal or contextual resources.

### 1.6 Psychological strain and resilience

Psychological strain typically reflects adverse psychological responses to stressors. However, resilience represents an adaptive capacity that enables individuals to recover or thrive in the face of adversity (Luthar et al., 2000). In competitive sports, exposure to high-pressure situations can lead to strain, but also may foster resilience when athletes perceive stress as a challenge rather than a threat (Jones et al., 2009). For instance, Fletcher and Sarkar (2012) highlighted that elite athletes often develop resilience by reframing stressors as opportunities for growth, which aligns with the Challenge- and Threat-Theory.

### 1.7 Coping humor as a moderator

Coping humor refers to the tendency to use humor as a means of managing stress (Martin et al., 2003). Prior research has demonstrated that humor can serve as a protective factor, buffering the adverse effects of strain and enhancing adaptive responses. For example, Abel (2002) found that individuals with high humor styles reported lower levels of perceived stress and greater psychological well-being. Similarly, Kuiper et al. (1993) showed that humor reduces negative affective responses to stressful situations.

Within moderation frameworks, coping humor is expected to influence how strain impacts resilience. When humor is frequently employed as a coping mechanism, individuals may perceive stressors as less threatening, enhancing resilience. Tugade and Fredrickson (2004) argued that positive emotions, such as those elicited through humor, facilitate psychological recovery and broaden cognitive resources, strengthening resilience. Empirical support also comes from Strick et al. (2009), who demonstrated that humor helps individuals regulate emotions and reduce stress reactivity, functioning as a moderator in stress adaptation processes.

### 1.8 Application in the current study

In this study, moderation analysis was conducted using PROCESS Macro Model 1 (Hayes, 2017), which allows testing the interaction effect between psychological strain and coping humor on resilience. The model creates an interaction term (X × W), and the statistical significance of this interaction supports the presence of moderation. Based on prior evidence, it was hypothesized that coping humor would strengthen the positive association between psychological strain and psychological resilience. This rationale is grounded in stress-coping theories (Lazarus and Folkman, 1984) and empirical findings demonstrating the protective effects of humor.

This study seeks to address the gap in understanding how humor functions as a coping mechanism in competitive sports by exploring the question:

“Does coping humor transform psychological strain into a source of resilience among professional football players?”

This research question directly aligns with the study’s purpose of examining the moderating role of humor-based coping in the relationship between psychological strain and resilience, aiming to clarify how humor contributes to athletes’ adaptive psychological functioning. In line with the specific demands of professional football, a high-pressure, team-oriented sport, the findings are expected to offer practical insights for psychological training programs designed to enhance athletes’ resilience, emotional regulation, and overall mental well-being through humor-based coping strategies. According to Simon et al. (2024), humor serves as a crucial psychological buffer, helping athletes cope with stress. Laughter and humor reduce cortisol levels and increase endorphin release, thereby alleviating athletes’ anxiety and tension and helping them recover more quickly after poor performance or failure. A humorous approach facilitates the reappraisal of challenging situations, decreases negative emotions, and strengthens team cohesion. In this sense, humor plays a significant role in developing both individual psychological resilience and collective team resilience (Simon et al., 2024).

Thus, the theoretical framework integrates psychological theories of resilience, stress appraisal, and coping humor, suggesting that athletes’ ability to use humor when under strain enhances their resilience in competitive contexts.

Grounded in this framework, it was hypothesized that psychological strain would exert a significant influence on athletes’ psychological resilience, and that coping humor would serve as a meaningful predictor of resilience levels. Furthermore, it was expected that coping humor would moderate the relationship between psychological strain and psychological resilience, such that the positive effect of psychological strain on resilience would become stronger as levels of coping humor increased. Specifically, when athletes display low levels of coping humor, psychological strain is not expected to have a significant association with resilience. At moderate levels of coping humor, strain is anticipated to significantly predict resilience, whereas at high levels of coping humor, the positive impact of strain on resilience is expected to be the strongest.

## 2 Materials and methods

### 2.1 Sample size estimation with Monte Carlo simulation (moderation analysis)

A Monte Carlo simulation method was used to determine the appropriate sample size for moderation analysis. This technique estimates statistical power when evaluating interaction effects, particularly under assumptions relevant to psychological research settings (Schoemann et al., 2017).

The simulation was conducted using the Monte Carlo Power Analysis tool, which was developed explicitly for this study. The analysis was based on the hypothesized model in which psychological strain (X) influences psychological resilience (Y), and this relationship is moderated by coping humor (W). In other words, the strength of the association between psychological strain and resilience is expected to vary depending on levels of coping humor.

The input values were:

Main effect of X (psychological strain → resilience) = 0.17.

Main effect of W (coping humor → resilience) = 0.18.

Interaction effect (X × W → Y) = 0.10.

*α* = 0.05.

Power = 0.80.

Number of bootstrap samples = 5,000.

Based on 10,000 replications, the analysis revealed that a minimum sample size of approximately 440 participants is required to achieve 80% power to detect the interaction (moderation) effect. The current study included 466 participants, which exceeds the recommended threshold, indicating sufficient statistical power for moderation analysis.

### 2.2 Population and sample

The population of this study comprises football players competing in professional leagues in Turkey during the 2024–2025 season. The inclusion criteria for participation were defined as follows: (i) being over 18 years of age; (ii) actively holding a license and playing for a team in the professional leagues during the 2024–2025 football season; (iii) being under 35 years of age; and (iv) voluntarily completing an informed consent form online. The convenience sampling method, a type of purposive sampling, was used for sample selection. This method allows researchers to collect data from accessible and willing participants and is frequently preferred in field studies (Yıldırım and Şimşek, 1999). The survey prepared for the research was transferred to the digital environment via Google Forms and distributed to players through club officials. At the end of the process, data were collected from 466 football players who met the inclusion criteria and voluntarily completed the informed consent form. Thus, the study sample consisted of 466 football players, selected voluntarily from professional league players across Turkey. The selection of football players as participants is theoretically grounded in the study’s conceptual framework, which focuses on the interaction between psychological strain, coping humor, and resilience in high-pressure athletic contexts. Football, as a team-based and highly competitive sport, presents constant psychological and social challenges, including performance anxiety, public scrutiny, and interpersonal stress within team dynamics. These factors make it an ideal context for examining how humor functions as a coping mechanism that transforms stress into resilience. Moreover, football players’ frequent exposure to intense physical and psychological demands provides a meaningful platform for exploring the adaptive value of humor and its role in maintaining mental well-being and consistent performance under pressure.

### 2.3 Study model

This correlational survey study investigates the relationships between psychological strain, psychological resilience, and coping humor in professional football players competing in top-level leagues. Furthermore, the study aimed to examine the moderating role of coping humor in the relationship between psychological strain and psychological resilience among professional football players.

Moderation effect analysis enables researchers to investigate whether the strength or direction of the relationship between two variables varies depending on the level of a third variable, known as the moderator (Hayes et al., 2017). In this framework, psychological strain (X) represents the independent variable, psychological resilience (Y) represents the dependent variable, and coping humor (W) functions as the moderating variable that influences the extent to which strain affects resilience.

Prior studies have emphasized that coping humor plays a protective and enhancing role in stress adaptation processes. For instance, humor has been identified as a psychological resource that strengthens resilience by reframing stressful experiences (Martin et al., 2003; Tugade and Fredrickson, 2004). Within sports, resilience is often developed through repeated exposure to high-pressure conditions, where coping strategies such as humor facilitate adaptation (Fletcher and Sarkar, 2012).

Accordingly, the present study hypothesized that coping humor moderates the effect of psychological strain on psychological resilience, such that the positive impact of strain on resilience becomes stronger at higher levels of coping humor.

**Figure 1:**
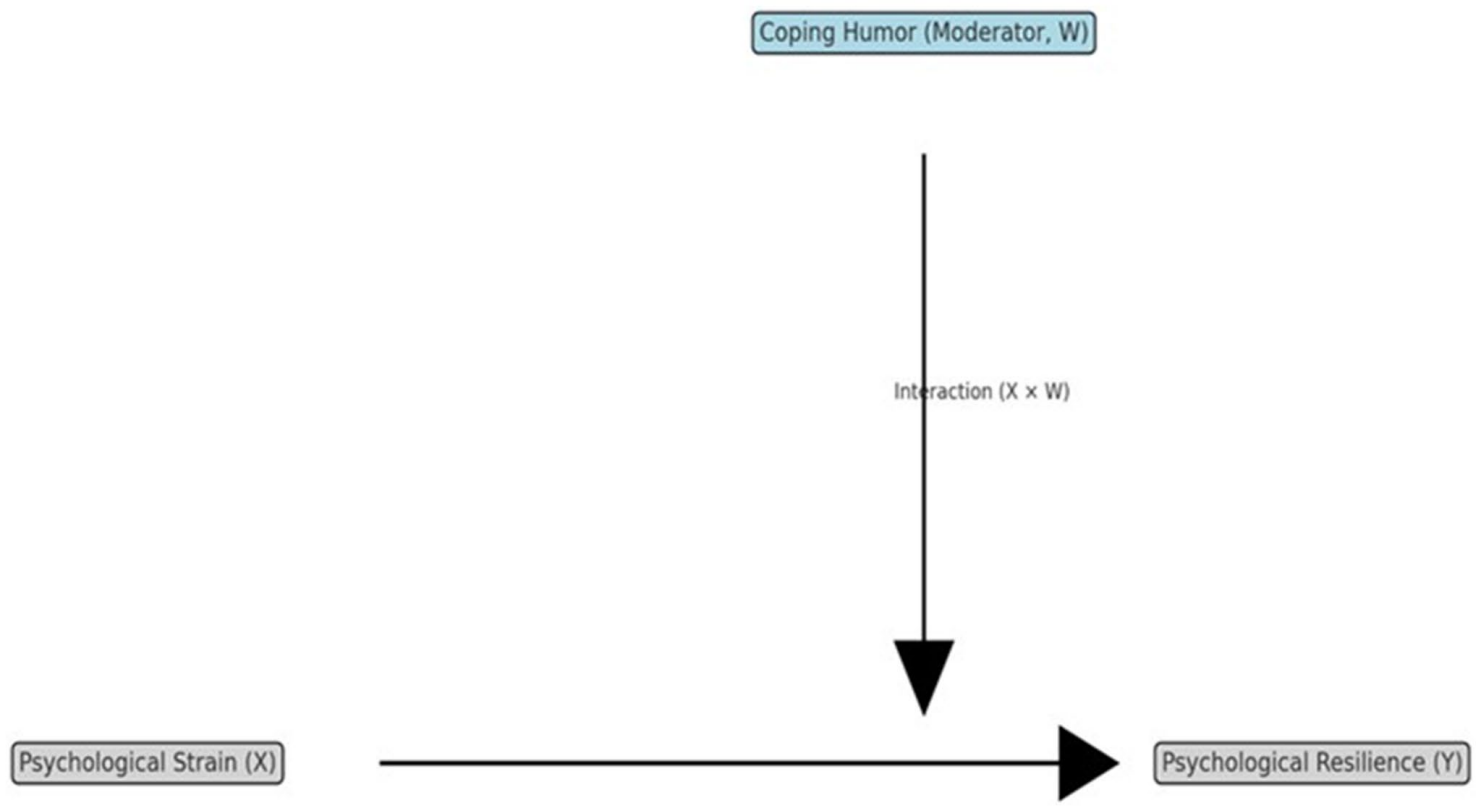
Model of the study.

Figure 1 Moderation model of the study: The effect of psychological strain (X) on psychological resilience (Y) moderated by coping humor (W).

Moderation analysis tests hypotheses suggesting that the causal effect between two variables is influenced by a third variable (Hayes et al., 2017). As shown in Figure 1, symbolic models of the study were drawn. In the symbolic models, Psychological Strain is defined as the primary independent variable; Coping Humor as the moderator variable; and Psychological Resilience as the dependent variable. The effect of Psychological Strain on Psychological Resilience was examined using simple regression analysis, as shown in Figure 1. The obtained result also tested Hypothesis 1 of the study. Whether Coping Humor moderates the relationship between the primary independent variable and the dependent variable was examined using PROCESS Macro Model 1. The PROCESS software automatically created an interaction variable (Psychological Strain × Coping Humor) by multiplying the primary independent variable and the moderator variable. Suppose the effect of the interaction variable on Psychological Resilience is significant. In that case, it indicates that Coping Humor moderates the causal relationship between the independent and dependent variables (Hayes et al., 2017). The obtained result also tested Hypothesis 2 of the study.

### 2.4 Data collection tools

Data collection for this study was conducted online using a form application. Despite their shared competitive grouping, this approach was favored due to its efficacy in reaching football players representing teams from different provinces. During the data collection phase, information about the study and questionnaires was sent to the participants through social networks. Participants were asked to complete a demographic information form developed by the researchers, as well as scales for psychological strain, psychological resilience, and coping humor from the literature, and a consent form confirming their voluntary participation in the study.

#### 2.4.1 Personal information form

In the present study, a 4-item information form, prepared by the researcher and consisting of questions regarding age, years of experience in football, the position played, and whether the participant had played at a professional level before, was administered to the participants.

#### 2.4.2 The athlete psychological strain questionnaire (APSQ)

The ASPQ was developed by Rice et al. (2020) to assess difficulties with team-based interactions, impaired impulse control and frustration tolerance, worries related to athletic performance and training stress, and the transition to life beyond professional athletic pursuits. It was subsequently adapted into Turkish by Lima et al. (2022). The scale has 10 items and three sub-dimensions (self-regulation difficulties, performance concerns, and external coping). Rice et al. (2020) reported that each item has a rating score from 1 to 5 (e.g., 1 = “None of the time”; 5 = “All of the time”). Participants’ scores of 15–16 on the total scale are considered moderate, 17–19 as high, and 20 and above as very high.

The Turkish version of the APSQ was adapted following the World Health Organization’s recommended translation–back translation procedure. Bilingual experts with experience in sports sciences conducted the translation process. Content validity was evaluated by three field experts, resulting in a Content Validity Index (CVI) of 0.90. The final Turkish version was reviewed and approved by the original scale developer, confirming that no additional linguistic or contextual modifications were necessary for its use with the Turkish athlete sample.

Lima et al. (2022) reported a Cronbach’s alpha coefficient of 0.83 for the total scale. The scale has been proven to have sufficient psychometric properties for widespread use, as it can assess the principal dimensions of psychological distress experienced by Turkish elite athletes (Lima et al., 2022). In this study, the scale was evaluated on total scores.

The Athlete Psychological Strain Questionnaire (APSQ) scale item 7: “I had a hard time coping with the pressures of being selected for the team.”

#### 2.4.3 The brief resilience scale (BRS)

BRS was developed to determine whether it is possible to reliably assess resilience as the ability to bounce back from stress, whether it is related to resilience resources, and whether it is related to important health outcomes. The version developed by Smith et al. (2008) and adapted into Turkish by Doğan (2015) was used. The scale has six items. Smith et al. (2008) reported that each item has a rating score from 1 to 5 (e.g., 1 = “Strongly Disagree”; 5 = “Strongly Agree”), and the second, fourth, and sixth questions are reverse-coded. It has been reported that high scores obtained from the scale indicate that the individual’s psychological resilience level is high.

The Brief Resilience Scale (BRS) item 3: “It does not take me long to recover from stressful situations.”

In the present study, the BRS was administered to an athlete sample. To ensure contextual appropriateness for this group, minor linguistic and contextual reviews were conducted. No substantive wording changes were required, as the original Turkish version was found to be clear and easily understandable for the participants. The items were therefore used in their original Turkish form, with instructions adapted slightly to refer to athletes’ experiences in training and competition contexts.

The Brief Resilience Scale was used to assess participants’ resilience levels. The Turkish version of the BRS has demonstrated good psychometric properties in university student samples, with a one-factor structure and an internal consistency coefficient (Cronbach’s *α*) of 0.83 (Doğan, 2015).

#### 2.4.4 The coping humor scale (CHS)

CHS is a self-report measure of different aspects of the sense of humor developed in investigating the stress-moderating effects of humor. The scale developed by Martin and Lefcourt (1983), later updated by Martin (1996) and adapted into Turkish by Yerlikaya (2009), was used in the study.

In this study, the Coping Humor Scale (CHS) was adapted into Turkish, and a comprehensive translation procedure was conducted to ensure linguistic and contextual equivalence. The scale was first translated into Turkish by the researcher and then reviewed by field experts for linguistic appropriateness. Following expert feedback, the Turkish version was back-translated into English and examined by the original developer of the scale, Prof. Dr. Rod Martin. Martin confirmed that the Turkish items preserved the original meanings, suggesting only minor linguistic adjustments. Subsequently, validity and reliability analyses were conducted on the Turkish form. As a result, both linguistic and contextual modifications were made in the Turkish version, achieving full cultural and semantic equivalence with the original scale.

The internal consistency coefficient (Cronbach’s α) of the Turkish version was reported as 0.67, and the test–retest reliability coefficient over a 12-week interval was 0.80 (Yerlikaya, 2009). In the present study, Cronbach’s α coefficients of all scales used were recalculated and reported in the results section.

The scale has seven items. Martin (1996) reported that each item has a rating score from 1 to 4 (e.g., 1 = “Strongly Disagree”; 4 = “Strongly Agree”). The total scores that can be obtained from the scale vary between 7 and 28, and higher scores indicate a higher tendency to use humor as a coping strategy in stressful situations.

The Coping Humor Scale (CHS) item 2. “I usually find that my problems become significantly smaller when I try to find something funny in them.”

### 2.5 Data analysis

The statistical analysis of the data used in the study was carried out through the SPSS v22 package program. In order to determine whether the data were normally distributed, it was checked whether the skewness and kurtosis values were within the ±2 value range (George and Mallery, 2016). To meet parametric assumptions for correlation and regression analyses, we tested the normality of each variable using the Shapiro–Wilk test and visual inspection through Q-Q plots. These tests indicated that the data did not deviate significantly from normality, allowing us to proceed with parametric methods (Field, 2024; Ghasemi and Zahediasl, 2012). As a result of the tests, it was seen that the data showed normal distribution and were found to be suitable for parametric tests. Confirmatory factor analyses of the scales were analyzed using the Jamovi 2.6.2.0 Package program (The Jamovi Project, 2024). Accordingly, the Pearson Correlation analysis was used to determine the correlations between the variables. The Fisher Z transformation test was applied to compare these relationships. Regression analysis was used to determine the effect of psychological strain on psychological resilience. To examine whether coping humor moderates the relationship between psychological strain and psychological resilience, a moderation analysis was conducted using PROCESS Macro v3.5 (Model 1) developed by Hayes (2013). Moderation analysis enables testing whether the effect of an independent variable (psychological strain) on a dependent variable (psychological resilience) changes depending on the level of a moderator variable (coping humor). In this analysis, the interaction term between psychological strain and coping humor was included in the regression equation, and its statistical significance was evaluated. The bootstrap resampling method with 5,000 samples was employed to obtain more robust estimates and confidence intervals. According to the bootstrap approach, moderation is considered significant if the 95% confidence interval of the interaction effect does not include zero (Hayes, 2013; Gürbüz, 2025).

## 3 Results

Table 1 shows that 36.5% of the football players were between the ages of 18 and 22, 34.5% were between the ages of 23 and 27, and 29.0% were between the ages of 28 and 35. The distribution of the players’ years of experience in football revealed that 15.7% were within the 5–10 years range, 41.8% were within the 11–15 years range, and 42.5% had an experience of 16 years and above. It was determined that 22.3% of the football players were goalkeepers, 26.0% were defensive players, 28.7% were midfielders, and 23.0% were offensive players. Additionally, 40.6% had played professionally before, while 59.4% did not.

**Table 1:** Descriptive statistics of the participants.

Variables	Groups	Frequency	Percentage (%)
Age	18–22	170	36.5
	23–27	161	34.5
	28–35	135	29.0
Years of experience in football	5–10	73	15.7
	11–15	195	41.8
	16+	198	42.5
Position you play	Goalkeeper	104	22.3
	Defence	121	26.0
	Mid-fielder	134	28.7
	Offensive	107	23.0
Played at the professional level before	Yes	189	40.6
	No	277	59.4

*N* = 466.

When Table 2 is examined, Cronbach’s Alpha values indicate that the internal consistency coefficient for the Athlete Psychological Strain Questionnaire is 0.895, for the Coping Humor Scale is 0.799, and for the Brief Resilience Scale is 0.851. These values demonstrate that the data provided by the participants on these scales exhibit an acceptable level of internal consistency.

**Table 2:** Reliability analysis results of the scales.

Scale	Number of items	Cronbach’s alpha
The athlete psychological strain questionnaire	10	0.895
The brief resilience scale	6	0.851
The coping humor scale	7	0.799

When Table 3 is examined, Confirmatory factor analysis (CFA) indicates that all three Measurement models demonstrated an excellent fit to the data. For the Athlete Psychological Strain Questionnaire, the fit indices were *χ*^2^/df = 2.36, CFI = 0.98, TLI = 0.97, RMSEA = 0.054, and SRMR = 0.028, all of which are within the acceptable to excellent range. The Brief Resilience Scale yielded *χ*^2^/df = 1.86, CFI = 0.99, TLI = 0.99, RMSEA = 0.043, and SRMR = 0.022, while the Coping Humor Scale demonstrated *χ*^2^/df = 1.56, CFI = 0.99, TLI = 0.99, RMSEA = 0.035, and SRMR = 0.022. These results suggest that the factor structures of the scales are well supported by the data, with all indices meeting or exceeding conventional criteria for good model fit (CFI and TLI ≥ 0.95, RMSEA ≤ 0.06, SRMR ≤ 0.08) (Hu and Bentler, 1999; Kline, 2023; McNeish et al., 2018).

**Table 3:** Fit indices of scales.

Scale	*χ*^2^/df	CFI	TLI	RMSEA	SRMR
The athlete psychological strain questionnaire	2.36	0.98	0.97	0.054	0.028
The brief resilience scale	1.86	0.99	0.99	0.043	0.022
The coping humor scale	1.56	0.99	0.99	0.035	0.022

Table 4 shows that the mean psychological strain value of the football players participating in the study was 21,863 ± 9,890, their mean coping humor value was 20,972 ± 3.980, and their mean psychological resilience value was 19,191 ± 3,925. It was also determined that the scale data showed a normal distribution.

**Table 4:** Descriptive statistics of the scales.

Scales	Min.	Max.	*X*	*SD*	Skewness	Kurtosis
Psychological strain	10.00	50.00	21.863	9,890	1.372	1.727
Coping humor	8.00	28.00	20.972	3.980	0.000	−0.383
Psychological resilience	13.00	30.00	19.191	3,925	1.155	1.386

The results of the Pearson Product–Moment Correlation Coefficient technique used to examine the relationships between all variables in the study are presented in Table 5.

**Table 5:** Pearson correlation coefficients for the correlations between the variables.

	1	2	3
1-Psychological strain	1	0.266^**^	486^**^
2-Coping humor	0.266^**^	1	0.298^**^
3-Psychological resilience	486^**^	0.298^**^	1

*N* = 466, **p < 0.01, 1-Psychological strain, 2-Coping humor, 3- Psychological resilience.

Table 5 shows that the psychological strain was positively and significantly correlated with coping humor (*r* = 0.266, *p* < 0.01), indicating that individuals who reported higher levels of psychological strain also reported a greater tendency to use humor as a coping mechanism. This finding supports the notion that humor may be employed adaptively in response to stress (Martin et al., 2003).

A moderate, positive, and statistically significant correlation was observed between psychological strain and psychological resilience (*r* = 0.486, *p* < 0.01). Although unexpected since strain is often associated with adverse outcomes, this result may be explained within the framework of the Challenge and Threat Theory (Jones et al., 2009). Athletes who perceive strain as challenging may engage in adaptive psychological responses, reinforcing their resilience.

Coping humor was also significantly and positively correlated with psychological resilience (*r* = 0.298, *p* < 0.01). This finding suggests that athletes who frequently use humor to cope with stress tend to exhibit higher levels of resilience. This is consistent with prior research identifying humor as a psychological resource enhancing stress tolerance and emotional recovery (Tugade and Fredrickson, 2004).

Table 6 shows the results of the simple linear regression analysis, which revealed that psychological strain significantly predicted psychological resilience among football players. The model was statistically significant, *F* (1,464) = 143.62, *p* < 0.001, indicating that psychological strain accounts for a meaningful portion of the variance in resilience.

**Table 6:** The effect of football players’ psychological strain on psychological resilience.

Variables								
Independent	Depend	β	*t*	*p*	*R*	*R* ^2^	*F*	*P*
Psychological strain	Psychological resilience	0.193	11.984	0.000	0.486	0.236	143.624	0.000

The standardized regression coefficient (*β* = 0.193, *t* = 11.98, *p* < 0.001) suggests a positive and statistically significant relationship between psychological strain and psychological resilience. This finding is counterintuitive, as psychological strain is typically expected to impact resilience negatively. However, in the context of competitive sports, this relationship may be explained through the Challenge and Threat Theory (Jones et al., 2009), which posits that when stress is perceived as a challenge rather than a threat, it can activate adaptive mechanisms such as resilience and mental toughness.

The model’s coefficient of determination, *R*^2^ = 0.236R^2 = 0.236*R*^2^ = 0.236, indicates that approximately 23.6% of the variance in psychological resilience can be explained by psychological strain. The correlation coefficient (*R* = 0.486) reflects a moderate positive relationship between the variables.

These results suggest that, in this sample of football players, higher psychological strain is associated with higher resilience, potentially due to their exposure to high-pressure environments that foster adaptive coping and mental fortitude.

**Figure 2:**
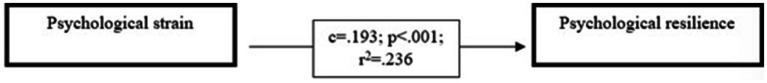
The effect of psychological strain on psychological resilience.

As shown in Figure 2, there is a significant and positive causal relationship between Psychological Strain and Psychological Resilience.

Table 7 examination of the collinearity statistics for the predictor variables indicates that the tolerance values for psychological strain and psychological resilience (0.764) are above 0.1, and the VIF values (1.310) are well below 10. These results suggest that there are no multicollinearity problems in the model.

**Table 7:** Collinetary statistics of predictor variables.

Variable	Tolerance	VIF
Psychological strain	0.764	1.310
Psychological resilience	0.764	1.310

VIF values below 10 and tolerance values above 0.1 indicate no multicollinearity problems.

As presented in Table 8, the Cook’s Distance values ranged from 0.000 to 0.052, with a mean (M) of 0.002 and a standard deviation (SD) of 0.005. These results indicate that no single observation had an excessive influence on the regression model. According to Cook and Weisberg (1982), Cook’s Distance values below 1.00 suggest the absence of influential data points. Therefore, the findings in Table 8 demonstrate that the regression model is stable and not unduly affected by any individual case.

**Table 8:** Cook’s distance statistics.

Statistic	Min	Max	X	SD
Cook’s distance	0.000	0.052	0.002	0.005

Table 9 presents the model summary results. The model explains approximately 10.8% of the variance in the dependent variable (*R*^2^ = 0.108), with an adjusted *R*^2^ of 0.104, indicating a modest but meaningful level of explanatory power. The correlation coefficient (*R* = 0.329) suggests a moderate positive relationship between the predictor variables and the outcome variable. The standard error of the estimate (3.766) indicates the average deviation of the observed values from the regression line. Importantly, the model was found to be statistically significant (*p* < 0.001), suggesting that the predictors collectively contribute significantly to the model.

**Table 9:** Model summary.

Model	*R*	*R* square	Adjusted *R* square	Std. error	Sig
1	0.329	0.108	0.104	3.766	0.000**

df1 = 2, df2 = 463, **p < 0.001.

Table 10 presents the results of the ANOVA analysis for the regression model. The analysis indicates that the model is statistically significant (*F* (2, 463) = 28.13, *p* < 0.001). The regression sum of squares (SS = 797.781) explains a substantial portion of the total variance (SS = 7364.637), while the residual sum of squares is 6566.856. This result demonstrates that the overall regression model provides a significantly better fit to the data than a model with no predictors, confirming that the independent variables collectively have a significant effect on the dependent variable.

**Table 10:** ANOVA analysis results for the regression model.

Model	Sum of squares	df	Mean square	Sig.
Regression	797.781	2	398.891	0.000^**^
Residual	6566.856	463	14.183	
Total	7364.637	465		

**p < 0.001.

As shown in Table 11, the regression analysis examined the predictors of coping humor. The results indicated that both psychological strain and psychological resilience significantly predicted coping humor. Specifically, psychological strain (*B* = 0.064, *β* = 0.159, *t* = 3.162, *p* = 0.002) had a positive and significant effect, suggesting that higher levels of psychological strain are associated with greater use of coping humor. Similarly, psychological resilience (*B* = 0.224, *β* = 0.221, *t* = 4.405, *p* < 0.001) was also a significant positive predictor, indicating that individuals with higher resilience tend to use coping humor more frequently. The model’s constant was statistically significant (*B* = 15.270, *t* = 17.505, *p* < 0.001), showing the baseline level of coping humor when predictor variables are held constant. Overall, these findings suggest that both strain and resilience contribute meaningfully to individuals’ use of humor as a coping strategy.

**Table 11:** Regression coefficients for predictors of coping humor.

Model	Unstandardized *B*	Std. error	Beta	*t*	Sig.
Constant	15.270	0.872	–	17.505	0.000**
Psychological strain	0.064	0.020	0.159	3.162	0.002*
Psychological resilience	0.224	0.051	0.221	4.405	0.000**

**p < 0.001, *p < 0.05.

PROCESS Macro Model 1. As shown in Table 12, the regression results presented in the table indicate that Psychological Strain significantly predicts Psychological Resilience (*b* = 1.182, *t* = 6.823, *p* < 0.001). In addition, Coping Humor also has a significant positive effect on Psychological Resilience (*b* = 0.566, *t* = 3.626, *p* < 0.001). Most importantly, the interaction term (Psychological Strain × Coping Humor) was also significant (*b* = 0.922, *t* = 6.845, *p* < 0.001), providing evidence that Coping Humor moderates the relationship between Psychological Strain and Resilience.

**Table 12:** Analysis results for determining the moderating role of coping humor.

Forecast variables		Psychological resilience			
		*b*	*SE*	*t*	*p*
Independent	Psychological strain	1.182	0.173	6.823	0.000***
Moderator	Coping humor	0.566	0.156	3.626	0.000***
Interaction effect	Psychological strain × Coping humor	0.922	0.135	6.845	0.000***
*F* = 77.432					
*R* = 0.578					
*R*^2^ = 0.335					
*p* = 0.000***					

*p < 0.05, ***p < 0.001, SE = Standard error. Standardized beta coefficients (b) are reported.

The overall model was statistically significant (*F* = 77.432, *p* < 0.001), with an explained variance of *R*^2^ = 0.335. This indicates that approximately 33.5% of the variance in resilience can be explained by strain, coping humor, and their interaction. The positive interaction effect suggests that the relationship between strain and resilience strengthens as coping humor increases.

These findings support the theoretical expectation that humor functions as a protective psychological resource. According to Martin et al. (2003), humor styles are associated with greater psychological well-being and adaptive coping. Consistent with the Broaden and Build Theory of positive emotions (Fredrickson, 2001), coping humor may broaden individuals’ thought-action repertoires, enabling them to find constructive solutions in stressful contexts. In turn, this strengthens resilience.

In the context of sport psychology, this aligns with Fletcher and Sarkar’s (2012) findings that elite athletes often rely on adaptive psychological strategies, such as humor, to transform stress into a performance-enhancing challenge. The significant moderation effect also resonates with the Challenge and Threat Theory (Jones et al., 2009), which posits that personal resources (such as humor) determine whether stressors are perceived as threats or challenges, influencing adaptive outcomes like resilience.

Thus, the table clearly demonstrates that coping humor has a direct, positive effect on resilience, amplifying the beneficial impact of strain on resilience. Athletes with higher humor-based coping skills are more likely to transform psychological strain into resilience, highlighting the moderating role of humor in stress adaptation processes.

Table 13 presents the conditional effects of psychological strain on psychological resilience at different levels of coping humor (low, medium, and high).

**Table 13:** The situational moderating effect of coping humor.

Coping humor level	*b*	*SE*	*t*	*p*	LLCI	ULCI
Low (−1)	0.262	0.263	0.994	0.321	−0.255	0.778
Medium (0)	1.189	0.173	6.878	0.000	0.849	1.528
High (+1)	2.116	0.165	12.815	0.000	1.791	2.440

At low levels of coping humor (−1 SD), the effect of strain on resilience was not significant (*b* = 0.262, *t* = 0.994, *p* = 0.321, 95% CI [−0.255, 0.778]). This indicates that strain does not significantly predict resilience for athletes who rarely use humor as a coping strategy.

At medium levels of coping humor (0 SD), the effect of strain on resilience was significant and positive (*b* = 1.189, *t* = 6.878, *p* < 0.001, 95% CI [0.849, 1.528]). This suggests that psychological strain begins to foster resilience when coping humor is used at an average level.

At high levels of coping humor (+1 SD), the positive relationship between strain and resilience was strongest (*b* = 2.116, *t* = 12.815, *p* < 0.001, 95% CI [1.791, 2.440]). This implies that athletes with high coping humor skills can transform strain into resilience.

The pattern indicates a moderation effect: the beneficial role of psychological strain in fostering resilience is contingent upon the level of coping humor. Specifically, coping humor enhances the positive impact of strain, with the most potent effects observed at high levels of strain.

This finding is consistent with the Broaden and Build Theory (Fredrickson, 2001), which posits that positive emotions (such as humor) broaden thought action repertoires, allowing individuals to cope more flexibly and recover from stress more effectively. Furthermore, Martin et al. (2003) emphasized that humor styles are adaptive coping mechanisms that protect psychological well-being.

In sports contexts, Fletcher and Sarkar (2012) demonstrated that elite athletes develop resilience by reframing stress as a challenge, and humor can serve as a key psychological resource in this reframing process. Similarly, Tugade and Fredrickson (2004) highlighted that resilient individuals use positive emotions to bounce back more effectively from stress, underscoring the moderating role of humor in the stress-resilience relationship. Thus, Table 8 demonstrates that coping humor is not just a buffer but an amplifier: the higher the coping humor, the stronger the positive effect of psychological strain on resilience.

**Figure 3:**
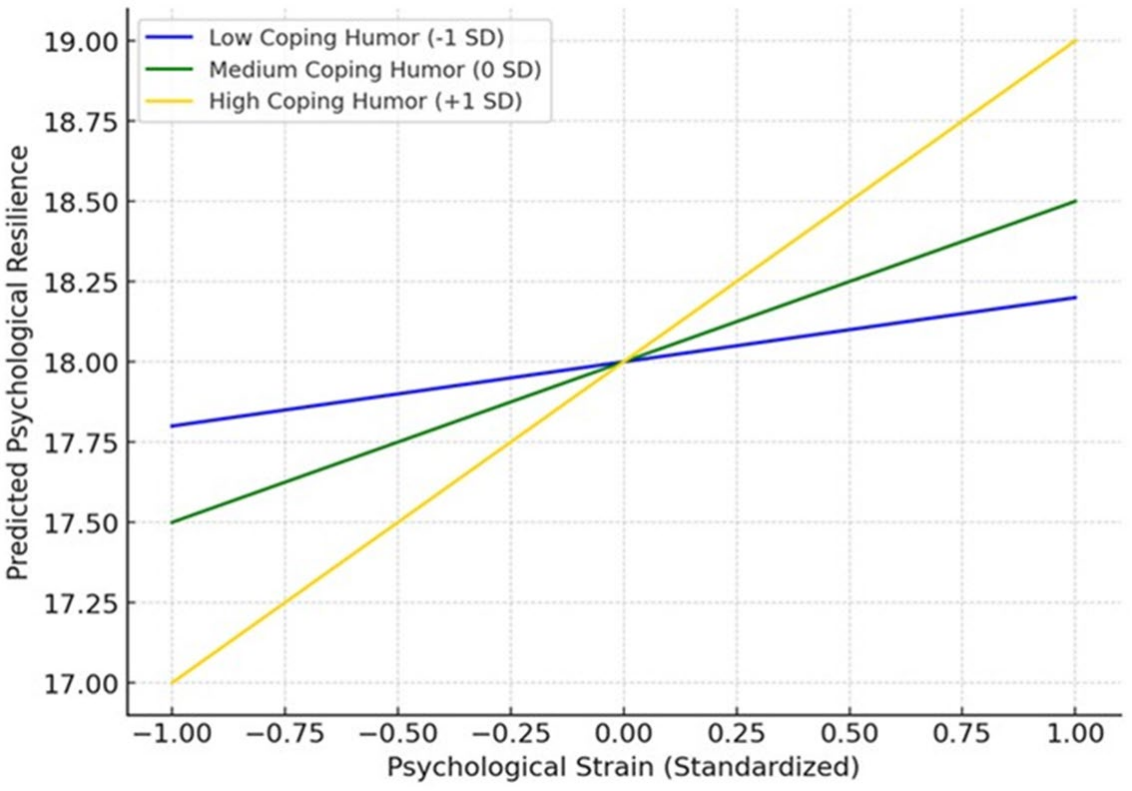
Conditional slope plot of coping humor in the effect of psychological strain on psychological resilience.

As shown in Figure 3, when participants’ levels of coping through humor are low, there is no moderating effect of Psychological Strain on Psychological Resilience. When participants’ levels of coping through humor are moderate, it plays a moderating role in the effect of Psychological Strain on Psychological Resilience, and this moderating effect increases as the levels of coping through humor rise.

It was determined that the levels of coping through humor (behaviors) in football players support the development of their psychological resilience, which enables them to cope with the stress generated by the physical, physiological, and psychological demands of the competition, and that it plays a positive regulatory role in their psychological performance under challenging conditions.

With this result, Hypothesis 2, stating that Coping Humor has a moderating role in the relationship between Psychological Strain and Psychological Resilience, is supported.

## 4 Discussion

The present study examined the relationships between psychological strain, coping humor, and psychological resilience among professional football players, with a particular focus on the moderating role of humor. The descriptive analysis of participants revealed a well-balanced distribution in terms of age, playing experience, and positions, providing a sound foundation for analyzing stress and resilience dynamics in competitive football environments.

Before interpreting the results, all statistical analyses were carefully reviewed to ensure analytical accuracy and transparency. Diagnostic checks confirmed that the regression models were stable and reliable, showing no signs of multicollinearity or influential outliers. The analyses demonstrated that the overall models were significant, and each predictor variable made a meaningful contribution. These findings reinforce confidence that the relationships among psychological strain, coping humor, and resilience accurately represent the underlying psychological mechanisms.

The results indicated that psychological strain had a positive relationship with resilience among athletes. In other words, athletes who experienced higher levels of stress tended to demonstrate stronger resilience. This finding aligns with Challenge and Threat Theory, which suggests that when stress is interpreted as a challenge rather than a threat, it can stimulate adaptive coping mechanisms that enhance mental toughness and resilience (Jones et al., 2009). Similar evidence from studies on elite athletes has shown that structured exposure to competition-related stress can strengthen coping skills and performance capacities (Fletcher and Sarkar, 2012). However, not all research has reported the same direction of effect; some studies have observed negative associations between stress and resilience (Doğan et al., 2024), which may be influenced by differences in culture, sport level, or the nature of stressors encountered. The assumption tests confirmed that these findings reflect stable and reliable relationships rather than statistical irregularities.

Coping humor also emerged as a significant positive predictor of resilience. Athletes who utilized humor as a coping mechanism demonstrated greater psychological strength, adaptability, and emotional balance when confronted with stress. This finding supports the view that humor serves as a psychological resource, promoting positive emotions, cognitive flexibility, and emotional regulation (Fredrickson, 2001; Levillain et al., 2024; Ashdown et al., 2025). Previous studies have similarly shown that athletes who use humor-based coping strategies tend to recover more quickly from stress and demonstrate higher mental toughness (Martin et al., 2003; Tugade and Fredrickson, 2004). Cultural norms regarding the expression of humor or team cohesion dynamics may influence the degree to which humor contributes to resilience (Nogueira et al., 2025).

Furthermore, the study found that coping humor moderated the relationship between psychological strain and resilience. Specifically, athletes with higher levels of coping humor benefited more from stress exposure, as humor amplified the positive influence of strain on resilience. This moderating effect is consistent with the Broaden and Build Theory (Fredrickson, 2001), which emphasizes that positive emotions expand individuals’ coping repertoires and enhance their ability to adapt to challenges. The results highlight that personal resources such as humor determine whether stress is perceived as a threat or an opportunity for growth (Jones et al., 2009; Fletcher and Sarkar, 2012). Parallel findings in sport psychology suggest that adaptive coping strategies, including humor, optimism, and social support, can transform stressful experiences into resilience-building opportunities (Tugade and Fredrickson, 2004; Sun et al., 2020).

Taken together, these results demonstrate that humor not only contributes directly to resilience but also strengthens the beneficial impact of stress. The robustness of the models and consistency of findings confirm that these effects reflect genuine psychological mechanisms rather than methodological artifacts.

The findings also expand current models of resilience and coping by suggesting that stress, when accompanied by adaptive personal resources such as humor, can catalyze growth rather than merely being a source of strain. This perspective supports a more dynamic and context-sensitive understanding of resilience, emphasizing that both the experience of stress and the appraisal of that experience shape psychological outcomes.

From a practical standpoint, the findings suggest valuable implications for sport psychologists and coaches. Integrating humor-based coping interventions into training routines may enhance athletes’ emotional regulation, team cohesion, and performance stability under pressure. Practices such as playful reframing, humor-oriented team-building, and targeted mental skills training could help athletes perceive strain as a manageable challenge rather than a debilitating threat (Çakmak, 2012; Thorson et al., 1997; Earvolino Ramirez, 2007). Training athletes to intentionally use humor as a reframing tool may also foster a more adaptive response to adversity.

Moreover, humor serves a dual function: it acts as a coping resource that reduces stress while simultaneously fostering social connectedness and team solidarity. Shared humor can build trust, increase motivation, and cultivate a sense of collective resilience within teams, reinforcing both individual and group-level psychological well-being.

In conclusion, the study provides compelling evidence that psychological strain can contribute positively to resilience when accompanied by effective humor-based coping strategies. Humor operates both as a direct enhancer of resilience and as a moderator that amplifies the beneficial effects of stress, underscoring its dual role in promoting psychological growth and adaptation. These findings, supported by rigorous analyses, highlight the importance of developing context-sensitive and strategy-focused approaches to resilience within the field of sport psychology (Fletcher and Sarkar, 2012; Levillain et al., 2024; Nogueira et al., 2025).

## 5 Conclusion

This study investigated the relationships between psychological strain, resilience, and coping humor among professional Turkish football players, with a focus on the moderating role of humor in the strain-resilience link. The findings provide significant theoretical, practical, and methodological insights.

### 5.1 Theoretical implications

The results confirmed that psychological strain positively predicts resilience, suggesting that stress in competitive sport can act as a challenge stimulus that facilitates adaptive growth rather than merely a threat. This aligns with the Challenge and Threat Theory (Jones et al., 2009) and previous findings by Fletcher and Sarkar (2012), highlighting that athletes who reinterpret stressors as challenges develop stronger resilience.

Furthermore, coping humor had a direct and significant effect on resilience. Humor serves as a protective psychological resource, facilitating emotional regulation, cognitive reframing, and maintaining a positive mindset under competitive pressure. This supports prior research indicating that positive affective strategies, including humor, enhance well-being and adaptive coping (Martin et al., 2003; Tugade and Fredrickson, 2004).

Crucially, humor moderated the relationship between strain and resilience. At low humor levels, the link between strain and resilience was nonsignificant; at moderate and high humor levels, strain was strongly associated with increased resilience. This finding supports the Broaden and Build Theory (Fredrickson, 2001), indicating that positive affect, such as humor, broadens cognitive and behavioral repertoires, enabling athletes to transform stressful experiences into opportunities for growth.

### 5.2 Practical implications

The findings provide actionable insights for coaches, sport psychologists, and athletes:

Integration of humor-based exercises: Mental training programs can include humorous reframing exercises, guided laughter sessions, and playful team-building tasks to enhance cognitive flexibility and reduce maladaptive stress responses.

Resilience-focused interventions: Humor can be effectively embedded into resilience workshops to help athletes reinterpret stressors as challenges, thereby improving psychological flexibility and enhancing performance consistency.

Structured reflective practices: Coaches may facilitate sessions where athletes share stressful experiences and reframe them in a humorous way, fostering a supportive team climate.

Screening and targeted support: Assessment tools can identify athletes who underutilize humor as a coping strategy, allowing tailored psychological support to prevent maladaptive stress responses.

Holistic mental training: Combining humor-based coping with mindfulness, goal-setting, or visualization can further strengthen resilience, emotional regulation, and overall performance.

### 5.3 Limitations

Several limitations of this study should be acknowledged:

Cross-sectional design: This design prevents causal inference regarding the relationships among psychological strain, coping humor, and resilience. Longitudinal or experimental studies would better establish temporal and causal mechanisms (Maxwell and Cole, 2007).

Self-report measures: Using only questionnaires may introduce common method bias or social desirability effects, particularly in athletic populations where psychological vulnerability is often stigmatized (Podsakoff et al., 2003).

Sample limitations: The study included only professional male football players in Turkey, limiting generalizability to female athletes, other sports, or different cultural contexts (Smith et al., 2010).

Humor styles not differentiated: Different humor styles (affiliative, self-enhancing, aggressive, self-defeating) can have distinct psychological effects (Martin et al., 2003), but were not examined in this study.

Other resilience predictors not included: Resilience is influenced by environmental, interpersonal, and cultural factors (Fletcher and Sarkar, 2012). This study focused only on psychological strain and humor, excluding variables such as social support, motivation, or coping flexibility.

Additionally, cultural specificity should be considered, as the findings reflect the unique sociocultural and sporting context of Turkish professional football, which may influence the expression of humor, stress perception, and resilience development. Therefore, future research should validate and extend these findings across diverse cultural and demographic samples using mixed-methods or cross-cultural approaches.

### 5.4 Future research directions

Future studies should:

Employ longitudinal or experimental designs to explore causal mechanisms through which coping humor influences resilience over time.

Include diverse samples, such as female athletes, participants from other sports, and different cultural contexts, to enhance generalizability.

Examine different humor styles to understand their distinct effects on psychological outcomes in sport.

Use multi-method approaches, incorporating physiological stress indicators (e.g., cortisol, heart rate variability) alongside self-report measures to reduce bias.

Investigate the interaction of humor with other psychological resources (social support, motivation, coping flexibility) to build a more comprehensive model of resilience in athletes.

Overall, the findings suggest that coping humor serves as both a direct enhancer of resilience and a moderator that transforms psychological strain into an adaptive stimulus. Integrating humor-based interventions into psychological training programs provides a practical, evidence-based approach to enhance resilience, regulate stress, and maintain high-level performance in athletes. These results emphasize the importance of positive affect, playful cognitive strategies, and holistic mental skills training as key components of athlete well-being and performance optimization. In practice, coaches, athletes, and sport psychologists can implement humor-based and resilience-enhancing strategies through structured workshops, reflective humor exercises, and team-based humor interventions designed to foster adaptive coping and group cohesion in sport environments. The methodological approach supports the robustness of these conclusions, as all measurement instruments (APSQ, BRS, and CHS) demonstrated acceptable reliability and construct validity in this sample. Additionally, the use of the PROCESS Macro with 5,000 bootstraps provided a statistically rigorous test of the moderation model. This methodological rigor strengthens the confidence in interpreting coping humor as both a direct and moderating factor within the resilience process. Future studies should explore causal mechanisms linking coping humor, resilience, and performance outcomes through longitudinal or experimental research designs, providing deeper insight into how humor operates as a dynamic resilience-building process in competitive sports.

## Data Availability

The original contributions presented in the study are included in the article/supplementary material, further inquiries can be directed to the corresponding authors.
